# A metabolic strategy to enhance long-term survival by Phx1 through stationary phase-specific pyruvate decarboxylases in fission yeast

**DOI:** 10.18632/aging.100682

**Published:** 2014-07-29

**Authors:** Ji-Yoon Kim, Eun-Jung Kim, Luis Lopez-Maury, Jürg Bähler, Jung-Hye Roe

**Affiliations:** ^1^Laboratory of Molecular Microbiology, School of Biological Sciences, and Institute of Microbiology, Seoul National University, Seoul 151-747;; ^2^University College London, Department of Genetics, Evolution and Environment, Darwin Building, Gower Street London WC1E 6BT, United Kingdom;; ^3^Current address: Instituto de Bioquímica Vegetal y Fotosíntesis, CSIC-Universidad de Sevilla, Avenida Américo Vespucio, s/n, 41092 – Sevilla, Spain

**Keywords:** Phx1, stationary phase, long-term survival, metabolic flux, pyruvate decarboxylases, ethanol

## Abstract

In the fission yeast *Schizosaccharomyces pombe*, the stationary phase-specific transcription factor Phx1 contributes to long-term survival, stress tolerance, and meiosis. We identified Phx1-dependent genes through transcriptome analysis, and further analyzed those related with carbohydrate and thiamine metabolism, whose expression decreased in *Δphx1*. Consistent with mRNA changes, the level of thiamine pyrophosphate (TPP) and TPP-utilizing pyruvate decarboxylase activity that converts pyruvate to acetaldehyde were also reduced in the mutant. Therefore, Phx1 appears to shift metabolic flux by diverting pyruvate from the TCA cycle and respiration to ethanol fermentation. Among the four predicted genes for pyruvate decarboxylase, only the Phx1-dependent genes (*pdc201^+^* and *pdc202^+^*) contributed to long-term survival as judged by mutation and overexpression studies. These findings indicate that the Phx1-mediated long-term survival is achieved primarily through increasing the synthesis and activity of pyruvate decarboxylase. Consistent with this hypothesis, we observed that Phx1 curtailed respiration when cells entered stationary phase. Introduction of *Δphx1* mutation compromised the long-lived phenotypes of *Δpka1* and *Δsck2* mutants that are devoid of pro-aging kinases of nutrient-signalling pathways, and of the *Δpyp1* mutant with constitutively activated stress-responsive kinase Sty1. Therefore, achievement of long-term viability through both nutrient limitation and anti-stress response appears to be dependent on Phx1.

## INTRODUCTION

Yeasts have served as good model systems to study aging and lifespan at a cellular level. When starved of nutrients, they start to reduce the rate of cell division and enter a resting state called stationary phase or quiescence [1]. In this state, the ability to adapt to nutrient limitation is crucial to sustain viability. Survival strategies involve physiological changes to reduce energy consumption by turning off mitotic cell division and declining protein synthesis, and at the same time to increase resistance to endogenous and environmental stresses. Since stationary phase cells can be viewed as post-mitotic cells under nutrient limitation, investigation of cellular strategies to maintain viability during stationary phase can provide information on ways to prolong chronological lifespan [2]. Two nutrient-sensing pathways, namely Tor/S6K and Ras/PKA pathways, which are activated primarily by amino acids and glucose, respectively, have been identified as proaging pathways in *Saccharomyces cerevisiae* (budding yeast) and *Schizosaccharomyces pombe* [3, 4]. These pathways are now known to be the major evolutionarily conserved pathways that sense the availability of nutrients and control chronological lifespan in various model organisms from yeasts to mammals [2, 5]. In *S. pombe*, activation of the stress-responsive MAP kinase (Sty1) pathway also prolongs chronological lifespan upon calorie restriction [6].

Whereas many studies revealed the involvement of signal transducing regulatory pathways in response to nutrients and stresses in determining lifespan, relatively little is known about physiological and molecular mechanisms that enhance survival during stationary phase. Some reported treatments that prolong chronological lifespan in yeasts include anti-oxidative means like overexpressing SOD [7] and mild heat stress [8]. This may reflect the physiological condition of stationary phase cells being under oxidative stress and with increased amounts of misfolded proteins that are susceptible to oxidative damage [1]. Work on *S. pombe* suggested oxidative stress in the stationary phase cells as a cause of death in glucose-rich media [9], although long-lived mutants are not necessarily more resistant to oxidative stress [10].

Caloric restriction by growth in low glucose media is known to contribute to long-term survival in various model organisms [5]. In *S. cerevisiae*, low glucose condition elevated respiration during the exponential phase, which in turn activated NAD^+^-dependent Sir2 deacetylase for lifespan extension [11]. In *S. pombe*, elevated respiration by low glucose during the exponential phase produced sufficient ROS to activate the Sty1 MAP kinase [6, 9]. A primary downstream transcription regulator, Aft1, which is activated by Sty1 kinase, induces expression of anti-stress genes that contribute to long-term survival in the stationary phase [9]. Unless Sty1 is activated during exponential phase, as observed in low glucose culture, stationary survival (chronological lifespan) will be compromised. Growth inhibition by drugs that target the Tor pathway also leads to extended chronological lifespan in *S. pombe* [12]. Some other genes were reported to affect lifespan with uncharacterized mechanisms. For example, overexpression of *ecl1+* gene that encodes a small nuclear protein was reported to increase chronological lifespan in *S. pombe*, whereas its deletion does not display any obvious phenotype [13]. Mutation in *rsv1^+^*gene encoding a zinc-finger protein was reported to lose viability during stationary phase [14].

Previously, we identified a stationary phase-specific regulator, Phx1, with a homeobox domain that positively affects long-term survival and meiosis upon nutrient starvation [15, 16]. The *phx1^+^* gene is expressed from late exponential to stationary phases, as well as in response to nutrient downshift. The *Δphx1* null mutant showed decreased viability in long-term stationary culture, and was more sensitive to various oxidants and heat shock. The *Δphx1/Δphx1* diploid cells were defective in forming meiotic spores. The target genes of Phx1 were expected to shed light on how Phx1 achieves these functions. In this study, we analyzedPhx1-dependent genes, and found a mechanism by which Phx1 contributes to chronological lifespan. It involves elevation of pyruvate decarboxylases to shift the carbohydrate/energy metabolism from respiration to fermentation as cells enter the stationary phase. This process presents a novel strategy to maintain viability by curtailing extended production of ROS in the stationary phase.

## RESULTS

### Effect of *Δphx1* mutation on stationary phase transcriptome of *S. pombe*

In order to understand how Phx1 enables long-term survival, stress resistance, and meiotic sporulation in *S. pombe*, we compared the global mRNA signatures of wild type vs. *Δphx1* deletion mutant cells by microarray analysis. Since the *phx1^+^* gene gets expressed from late exponential phase, and the viability of *Δphx1* cells starts to decrease rapidly from ~3 days after entering stationary phase (~100 h post-inoculation in EMM; [15]), we prepared RNA samples at 80 h culture time when cells still retained full viability. RNAs from four independent cultures were subjected to cDNA synthesis and hybridization. The microarray analysis revealed that transcripts from 56 genes were decreased by more than 2-fold and expression levels of 97 genes were increased by more than 2-fold in the *Δphx1* mutant compared with wild type (Supplementary Table S1, S2). We summarized the affected genes with functional grouping by generic GO-term finder (http://go.princeton.edu/cgi-bin/GOTermFinder) in Table 1 and Table 2 for genes positively and negatively affected by Phx1, respectively.

**Table 1:** Functional categories of 56 genes lower expressed in *Δphx1* mutant

Function	Number of genes	Gene ID or Name
thiamine and derivatives biosynthetic process	2	nmt1, nmt2
carbohydrate metabolic process	5	eno1, gpd3, dak2, SPAC13A11.06 (pdc202), SPAC3G9.11c (pdc201)
response to stress	7	SPAC869.09, zym1, SPAC22G7.11c, SPBC8E4.05c, SPAC11D3.01c, taf1, hsp16
transport	5	cta3, SPAC977.17, bsu1, mfs1, SPCPB1C11.03
RNA metabolic process	2	aes1, SPBC530.08
non-coding RNA	7	prl26, SPNCRNA.74, SPNCRNA.101, SPNCRNA.73, prl01, SPNCRNA.79, prl12
vesicle-mediated transport	4	SPAC824.02, glo3, SPAC3C7.02c, imt2
protein phosphorylation	2	ppk31, crk1
cytoskeleton organization	1	SPBC1289.14
oxidation-reduction	3	SPBC1198.01, but1, SPAC5H10.04
others	18	SPAC869.06c, SPAPB18E9.04c, SPBPB21E7.02c, SPBPB21E7.04c, SPAC1F7.06, SPAPB18E9.03c, SPBC19C7.04c, SPAC4F10.17, SPAC1093.01, SPAC11D3.02c, SPAC15F9.01c, SPAC9E9.01, SPAC30D11.02c, SPBC530.07c, SPCPB16A4.06c, mug138, SPCC417.12, SPAC1142.01

**Table 2:** Functional categories of 97 genes higher expressed in *Δphx1*

Function	Number of genes	Gene ID or Name
**Function**	**Number of genes**	**Gene ID or Name**
carbohydrate & energy metabolism	19	agl1, inv1, SPAC1039.11c, cyc1, cit1, aco1, SPAC3A11.07, SPAC26H5.09c, SPAC3C7.13c, zwf1, SPAC4G9.12, SPACUNK4.10, gut2, SPAC9E9.09c, psd2, tms1, erg25, SPCC191.05c, SPBC800.11
response to stress	12	rds1, tos4, SPBC660.05, SPBC1271.08c, SPCC1739.08c, hri1, SPACUNK4.15, SPAC27D7.11c, sod1, srx1, SPAC11D3.16c, alo1
conjugation / meiosis	13	ste7, mfm1, rgs1, isp4, mei2, mfm2, mfm3, map2, isp7, ste11, ogm1, rep1, mam1
transport	22	ght3, ght4, ght5, ght8, fip1, fio1, SPBC947.05c, ptr2, SPBC16A3.02c, SPCC794.03, SPCC569.05c, SPBC1348.05, SPAC750.02c, SPBPB2B2.16c, SPAC323.07c, vht1, bfr1, anc1, crp79, SPBC530.02, abc3, atp2
non-coding RNA	6	prl3, SPNCRNA.93, SPNCRNA.133, SPNCRNA.134, prl7, SPNCRNA.63
others	25	SPAC186.05c, SPAC977.07c, SPAC186.04c, SPAC1A6.03c, mik1, SPAC212.03, SPAPB18E9.05c, SPAC977.04, SPAC977.05c, SPAC977.02, SPCC584.16c, SPAC513.04, SPBC359.06, SPAPB15E9.01c, SPBC1348.04, SPAC977.03, SPBC9B6.03, SPCC553.10, SPCC1450.07c, SPBPB2B2.19c, SPBC1348.03, arg7, SPAC977.01, SPAC27E2.04c, SPAC24C9.08

The genes positively affected by Phx1 (or repressed in the *Δphx1* mutant) include those known or predicted to function in thiamine biosynthesis, carbohydrate metabolism, stress responses, transport, RNA metabolic process, and non-coding RNA (Table 1, Table S1). Several genes have functions related to thiamine metabolism. Prominent examples are the *nmt1^+^* and *nmt2^+^* genes that encode biosynthetic enzymes of pyrimidine and thiazole moiety of thiamine, respectively [17]. The *bsu1^+^* (*car1^+^*) gene encodes a transporter for pyridoxine derivatives and thiamine [18]. Two genes (SPAC13A11.06 and SPAC3G9.11c) encode putative pyruvate decarboxylases that require thiamine pyrophosphate (TPP) as a cofactor. Therefore, it appears that Phx1 contributes to increasing the level of thiamine and TPP-requiring enzyme pyruvate decarboxylase that converts pyruvate to acetaldehyde.

The genes negatively affected by Phx1 (or induced in *Δphx1*) also involve many genes for carbohydrate and energy metabolism including TCA cycle and pentose phosphate pathway, stress response, conjugation and meiosis, and transport (Table 2, Table S2). Prominently, genes for mitochondrial energy generation such as cytochrome c (*cyc1^+^*), citrate synthase (*cit1^+^*), aconitase (*aco1^+^*), mitochondrial glycerol-3-phosphate dehydro-genase (*gut2^+^*), and a putative NADH dehydrogenase (SPAC3A11.07) were negatively regulated by Phx1. These results suggest that Phx1 inhibits mitochondrial energy generation. We also noticed that genes predicted for the pentose phosphate pathway such as glucose-6-phosphate dehydrogenase (*zwf1^+^*, SPAC3C7.13c), glucokinase (SPAC4G9.12), and the *gfo/idh* family oxidoreductase (SPAC26H5.09c) were induced in *Δphx1* mutants, possibly reflecting that the mutant cells are under oxidative stress. This finding coincides with the observation that several genes involved in oxidative stress response are induced in the mutants, such as *sod1^+^*,encoding CuZnSOD, and *srx1^+^*, encoding sulfiredoxin. Several transporter genes forhexose (*ght3^+^*, *ght4^+^*, *ght5^+^*, *ght8^+^*) were also induced in *Δphx1* mutants. Considering the previous observation that *ght3^+^* and *ght4^+^* genes are induced under oxidative and heat stress conditions [19], it is possible that *Δphx1* cells experience more oxidative stress than wild type cells.

Many genes whose expression is correlated with different phases of meiotic differentiation were affected by Phx1. Among the previously reported meiosis-correlated genes [20], 21 genes were repressed in the Δ*phx1* mutant and 40 genes were induced (Table S1). It is likely that the mis-regulation of these genes lie behind the sporulation-deficient phenotype of the Δ*phx1/*Δ*phx1* diploid mutant [15]. Inspection of the affected meiotic genes revealed that Phx1 primarily up-regulated middle and late meiotic genes, functioning during meiotic divisions and sporulation (16 out of 21 genes; Table S1), and down-regulated genes of early meiotic genes, functioning during nitrogen starvation and meiotic prophase (27 out of 40 genes; Table S2). It can be hypothesized that Phx1 is a major regulator that activates later phases of meiotic gene expression (such as meiotic divisions I, II, and spore formation)and represses genes of earlier meiotic stages (such as starvation response, pheromone sensing, conjugation, S phase and DNA recombination). How Phx1 works in concert with other known regulators of late meiotic stages, such as Rsv1, Rsv2, Atf21 and Atf31 [21], will be an interesting topic to investigate in the future.

### Phx1 functions to maintain the thiamine pool during stationary phase

We further examined whether thiamine-metabolic genes are indeed regulated by Phx1. Particularly, the possibility of feedback inhibition of thiamine biosynthetic (*nmt1^+^* and *nmt2^+^*) and transport (*bsu1^+^*) genes by thiamine in the *Δphx1* mutant needed to be evaluated. For this purpose, we monitored the mRNA levels of *nmt1^+^*, *nmt2^+^*, and *bsu1^+^* genes along with the *thi9^+^* gene, encoding a thiamine transporter [18], which is thiamine-repressible but not affected by Phx1. Transcripts from the *tnr3^+^* gene encoding thiamine diphosphokinase [22], which is not repressible by thiamine, were monitored in parallel as a control.

**Figure 1:**
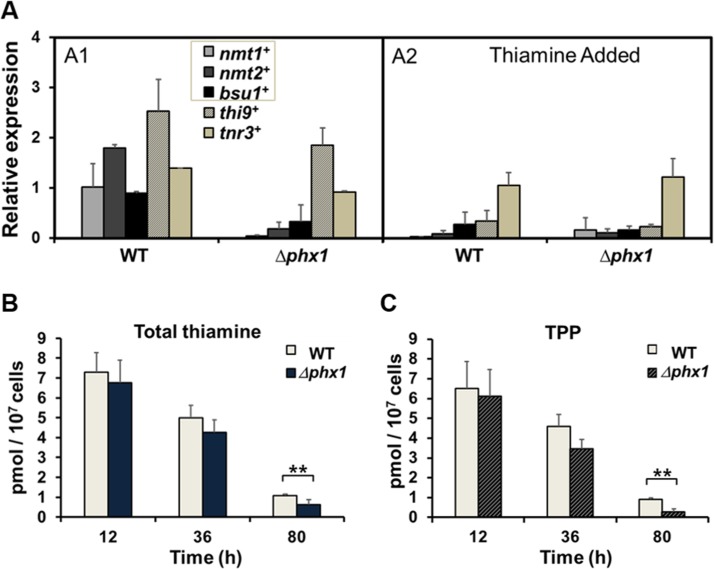
Thiamine supply is activated by Phx1 (**A**) The mRNA levels of genes involved in thiamine biosynthesis (*nmt1^+^*, *nmt2^+^*), transport (*bsu1^+^*, *thi9^+^*), and metabolism (*tnr3^+^*) in wild type (WT; JH43) and *Δphx1*(ESX5) mutant. Cells were grown in minimal media to either exponential or stationary phases, with (A2) or without (A1) adding 10 μM thiamine. The gene-specific mRNA levels were measured by qRT-PCR, along with that of *act1^+^* mRNA as an internal control. Each internally normalized expression level at stationary phase was presented in the figure as a relative value to the level in exponential cells. Average values from three independent experiments were presented with standard deviations. (**B, C**) Intracellular levels of total thiamine pool (**B**) and TPP (**C**). Wild-type and *Δphx1* mutant cells grown in minimal media were harvested at early exponential (12 h), late exponential (36 h), and late stationary (80 h) phases. Thiamine and thiamine phosphates (TMP, TPP) were extracted and measured by HPLC. Total thiamine is the sum of thiamine and thiamine phosphates. Average values with standard deviations (error bars) from at least three independent experiments were presented. Asterisks (**) represents p-value of <0.05 in Student *t-test*.

Results from qRT-PCR of RNAs from wild-type and *Δphx1* mutant cells demonstrated that the*nmt1^+^*, *nmt2^+^*, and *bsu1^+^* transcripts decreased in the *Δphx1* mutant in the absence of thiamine, whereas the *thi9^+^* gene was not, as observed in microarray analysis (Fig. 1, panel A1). Added thiamine repressed expression of all genes, except *tnr3^+^* in the wild type (Fig. 1, panel A2). The *thi9^+^* gene was also repressed by thiamine in the *Δphx1* mutant (Fig. 1, panel A2). Therefore, the decrease in the expression of three Phx1-dependent genes (*nmt1^+^*, *nmt2^+^*, *bsu1^+^*) is not due to feedback regulation by increased thiamine in the *Δphx1* mutant, but suggests that Phx1 positively regulates the three genes for thiamine supply.

We then determined the intracellular thiamine pool. The amounts of thiamine, thiamine monophosphate (TMP), and thiamine pyrophosphate (TPP) were measured by HPLC in cell-free extracts obtained at different growth phases. As shown in Fig. 1B, the total thiamine pool decreased as cells progressed into stationary phase. The decrease, however, was more pronounced in the Δ*phx1* mutant, where the total thiamines were reduced to about 60% of wild type level during stationary phase (80 h culture). The majority of the thiamine pool consisted of TPP, which is the active form as an enzyme co-factor, consistent with previous data [17]. The Δ*phx1* mutant contained a much lower amount of TPP, only about 30% of the wild-type level during the stationary phase (80 h; Fig. 1C). The ratio of TPP to thiamine was maintained from 8.4 to 5.5 in the wild type from exponential (12 h) to late stationary phase (80 h) cells, whereas the ratio changed from 9.4 to 0.8 in the mutant during the same period. The amount of TMP was below our detection limit. Overall, these measurements demonstrated that Phx1 is needed to maintain the level of thiamine pool, especially TPP, during stationary phase.

### Phx1 is needed for the synthesis of stationary phase-specific pyruvate decarboxylases

**Figure 2:**
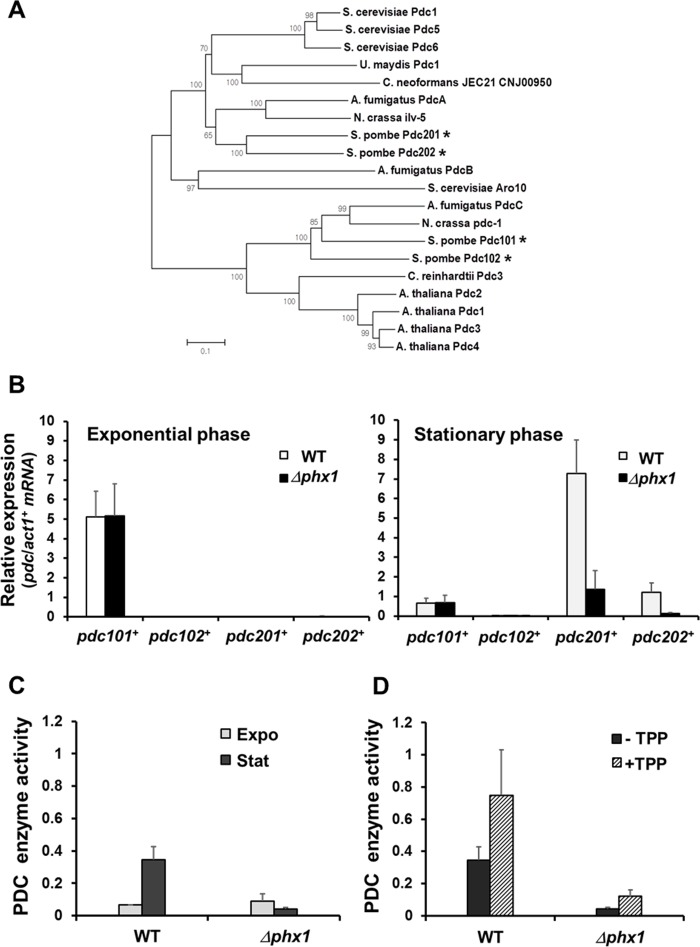
Stationary phase-specific pyruvate decarboxylases are regulated by Phx1 (**A**) The phylogenetic relatedness of various fungal PDC proteins. Amino acid sequences were aligned with ClustalW program, and a phylogenetic tree was constructed using the Neighbor-Joining method in MEGA 5 program. A Bootstrap test was performed for 1000 replicates and the values were indicated at each node. (**B**) Expression levels of *pdc101^+^, pdc102^+^, pdc201^+^*, and *pdc202^+^* genes in the wild type (JH43) and *Δphx1* mutant (ESX5) at two growth phases. RNA samples were obtained from cells grown in EMM for 18 and 50 h for exponential and stationary phase cultures, respectively. The amounts of gene-specific mRNAs were estimated by qRT-PCR, along with that of *act1^+^* mRNA as an internal control. Relative expression values to *act1^+^* mRNA were obtained from three independent experiments, and were presented as an average with standard deviations. (**C**) Phx1-dependent PDC enzyme activity. Cell extracts were obtained from cells as described in (**B**). Pyruvate decarboxylase activity was measured as described in the text. Average values from three independent experiments were presented with standard deviations. (**D**) Effect of TPP addition on PDC activity. Experiments were done as in (**C**), except that TPP was added at 100 μM (final) to cell extracts.

The microarray analysis revealed that transcripts from two genes (SPAC13A11.06 and SPAC3G9.11c) encoding putative pyruvate decarboxylases (PDCs) were significantly lowered in *Δphx1* mutant (Table 1, Table S1). The PDC enzyme utilizes thiamine pyrophosphate (TPP) as a cofactor, and constitutes a distinct subgroup of the TPP-dependent enzyme family [23]. There are four PDC-like proteins predicted from the genome of *S. pombe*. Since the gene name *pdc1* has been already used for mRNA decapping scaffold protein (SPAC4F10.01) [24], we propose to name the paralogs as *pdc101*(SPAC1F8.07c), *pdc102*(SPAC186.09), *pdc201*(SPAC3G9.11c), and *pdc202*(SPAC13A11.06). Phylogenetic relatedness of PDC-like proteins in *S. pombe* as well as in other representative fungi is presented in Fig. 2A.

The Pdc201 and Pdc202 cluster together, along with three reported PDCs of *S. cerevisiae* (ScPdc1, 5, 6; [25], two PDCs of basidiomycota (*U. maydis*, *C. neoformans*), PdcA of *Aspergillus fumigatus* and Ilv-5 of *Neurospora crassa*, and more distantly with ScAro10 (YDR380w) and AfPdcB (Afu6g00750). ScAro10 was reported to be involved in the degradation of aromatic amino acids as a phenylpyruvate decarboxylase [26]. Pdc101 and Pdc102 clustered closely in a separate clade, along with PDCs of *N. crassa* and *A. fumigatus* and four PDC proteins from plant kingdom such as *Arabidopsis thaliana* and *Chlamydomonas reinhardtii* (Fig. 2A).

We monitored the expression profiles of four *pdc* genes during growth and stationary phases. Each mRNA level was determined by qRT-PCR, and presented as a normalized value relative to the control *act1^+^* mRNA (Fig. 2B). The results demonstrate that *pdc101*^+^ is the predominant gene in the exponential phase, whose expression decreases during stationary phase. Therefore, Pdc101 is most likely the primary pyruvate decarboxylase that supports exponential growth. Expression of *pdc201^+^* and *pdc202^+^* genes increased markedly during stationary phase, by about 500-fold and 50-fold, respectively. The *pdc102^+^* gene produced hardly detectable mRNA in both conditions. Expression of *pdc201^+^* and*pdc202^+^* genes was dependent on Phx1, whereas that of *pdc101^+^*was not (Fig. 2B). Taken together, we found that *pdc201^+^* and *pdc202^+^* genes are induced highly during the stationary phase and their induction is mediated by the positive regulator Phx1.

We then determined whether Phx1 indeed controls the level of PDC enzyme activity. Results in Fig. 2C demonstrated that PDC enzyme activity increased during stationary phase in wild type, but decreased in the mutant. PDC activity at the exponential phase was not affected by *phx1* mutation. Therefore, consistent with the transcript regulation, Phx1 indeed controls the level of stationary phase-specific PDC enzymes. Since Phx1 controls the synthesis of both PDC apo-proteins and its cofactor TPP,we investigated the effect of adding thiamine to a stationary phase cell culture. Results in Fig. 2D show that added thiamine increased activities of PDCs in the wild type and the mutant, suggesting that TPP can be a limiting factor for PDC activity during stationary phase. However, thiamine addition did not fully elevate the PDC activity to wild type level,confirming that the *phx1* mutation indeed significantly decreased the level of PDC proteins during stationary phase. Therefore, our results revealed that Phx1 positively regulates the supply of thiamine pyrophosphate as well as the production of Pdc201 and Pdc202 proteins, leading to increase in PDC enzyme activity during stationary phase. PDC diverts pyruvate from being used in mitochondria for the TCA cycle and respiration to the direction of ethanol fermentation.We hypothesize that this metabolic shift could be a mechanism behind the cellular strategy to maintain viability during stationary phase.

### Pdc201 and Pdc202 contributes to long-term survival of *S. pombe*

**Figure 3:**
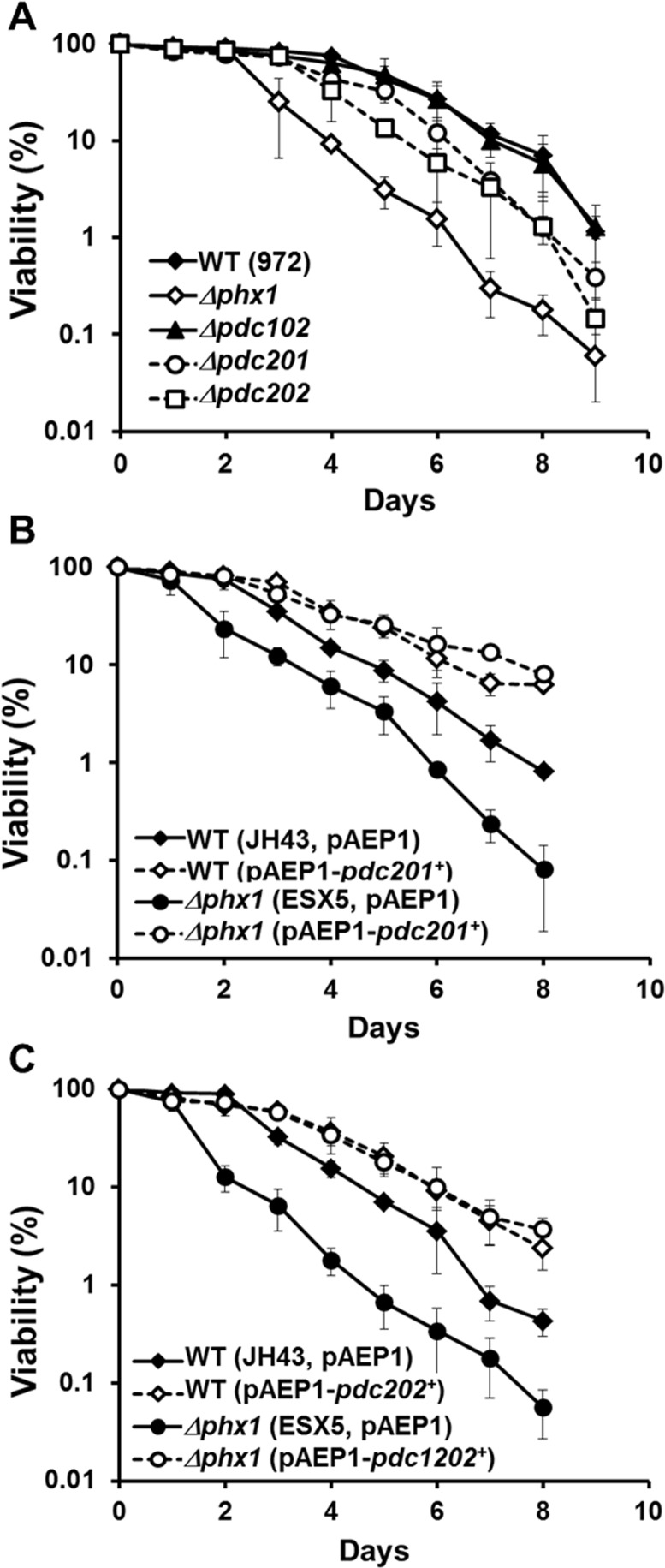
Pdc201 and Pdc202 contribute to long-term survival (**A**) Long-term survival of the wild type (WT; 972), *Δphx1* (JY01 in 972 background), *Δpdc201, Δpdc202*, and *Δpdc102* mutants. Viability assay was done as described in the text. At least three independent experiments were carried out to obtain survival curves for each strain. (**B**) Effect of overproducing Pdc201. Viability of wild type (JH43) and *Δphx1* mutant (ESX5 in JH43 background) cells with pAEP1-*pdc201^+^* plasmid or parental plasmid were measured as in (**A**). (**C**) Effect of overproducing Pdc202. Viability of wild type (JH43) and *Δphx1* (ESX5) cells containing pAEP1-*pdc202^+^* plasmid or parental plasmid were measured.

Whether PDC contributes to long-term survival of *S. pombe* was assessed by gene disruption and overexpression. We created Δ*pdc201*, Δ*pdc202*, and Δ*pdc102* mutants and compared their long-term viability in EMM with the wild type (972) and the *Δphx1* mutant (JY01). We were not able to obtain a *Δpdc101* mutant, consistent with the prediction that this gene is essential [27]. The results in Fig. 3A demonstrated that the Δ*pdc201* and *Δpdc202* mutations reduced cell viability in the stationary phase, even though not as pronounced as the effect of *Δphx1* mutation. The *Δpdc102* mutation did not affect cell viability significantly. The double mutation of *Δpdc201Δpdc202* did not cause further reduction in viability (supplementary Fig. S1). We then examined the effect of overproduction by cloning the *pdc201*^+^ or *pdc202*^+^ gene behind the *adh1^+^* promoter on pAEP1, a pREP1-based vector [16], and introduced them to the wild type (JH43) or *Δphx1* (ESX5). Results in Fig. 3B and 3C demonstrated that overproduction of either Pdc201 or Pdc202 increased the long-term viability of both the wild type and *Δphx1* mutant. These observations support the proposal that the mechanism behind the positive action of Phx1 in extending stationary cell survival relies heavily on the increase in pyruvate decarboxylases.

### Phx1 decreases mitochondrial respiration and ROS production, and increases ethanol fermentation upon entering into stationary phase

**Figure 4:**
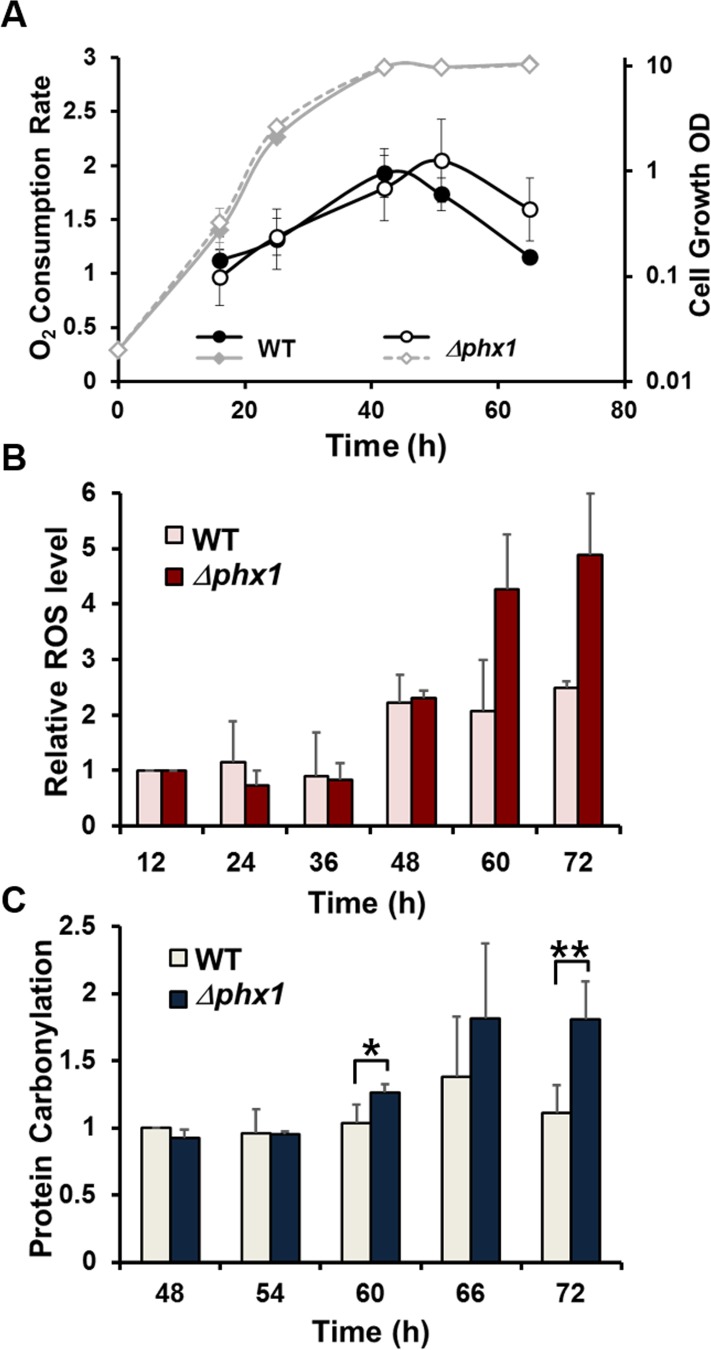
Oxygen consumption, ROS accumulation, and protein oxidation during growth. (**A**) Oxygen consumption rate throughout cell growth. The wild type (JH43) and *Δphx1* (ESX5) mutant cells were inoculated to an initial OD600 of 0.02 in EMM, and monitored for growth at 30°C by measuring OD_600_ (diamonds). Aliquots were taken at different growth phases, and measured for oxygen consumption rates (circles). Average values with standard deviations were obtained from three independent experiments, and presented as %O_2_ consumed per ml of cell culture per min per OD600 to normalize the amount of cells in each sample. (**B**) Relative levels of intracellular ROS. Cells were grown in the same way as in (**A**). At the indicated time points, aliquots were taken and mixed with 2′,7′-dichlorofluorescin diacetate (DCFH-DA). Fluorescence from 10,000 cells for each sample was monitored by flow cytometry. Values relative to the fluorescence from wild type and *Δphx1* cells at 12 h were presented for the rest of the samples. Average values with standard deviations were obtained from three independent culture samples. (**C**) Relative levels of protein carbonylation. Cells were grown in the same way as in (**A**). Carbonylated proteins were detected with anti-DNP antibodies. The carbonylation level of wild type cells at 48 h was taken as 1, and relative values were presented for other samples. Average values with standard deviations were obtained from three independent culture samples. * and ** represent p-values of <0.1, and <0.05, respectively, in Student *t-test*.

Based on the increased synthesis and activity of Pdc201 and Pdc202 enzymes during stationary phase, we hypothesized that mitochondrial respiration may decrease as *S. pombe* cells enter into stationary phase. We therefore estimated oxygen consumption rate in the wild type and the *Δphx1* mutant grown in minimal media. Fig. 4A demonstrated that the oxygen consumption in the wild type cells peaks at around 40 h after inoculation, when cells enter the stationary phase, and decreases steadily thereafter. On the other hand, the Δ*phx1* mutant maintained the high rate of respiration longer, for about 20 hours after cells entered stationary phase. When we measured glucose consumption during the growth of wild type and Δ*phx1* mutant cells, we found that glucose was exhausted at around 40 h, and there was no difference between wild type and Δ*phx1* mutant in consuming glucose (data not shown). This implies that the Δ*phx1*mutation does not affect sugar metabolism, and possibly energy metabolism, during the exponential growth.

Since mitochondrial respiration is considered to be a major source of reactive oxygen species (ROS) whose accumulation damages cell components and hence could curtail lifespan, we examined the amount of intracellularROSproduction by a redox-sensitive fluorescent dye: 2′,7′-dichlorofluorescin diacetate (DCFH-DA). Fig. 4B demonstrated the level of DCFH-oxidizable ROS in the wild type and *Δphx1*cells grown for different lengths of time in minimal media as in Fig. 4A. The fluorescence of individual cells was measured by flow cytometry, and the intensity from 10,000 cells was compared relative to the value at 12 h culture. The results indicated that the*Δphx1* mutant accumulated higher amount of ROS than the wild type during stationary phase (60, 72 h culture time), when *Δphx1* mutant cells still retain full viability in minimal media [15]. This coincides with the prolonged respiration observed in the mutant. We further examined whether the *Δphx1*cells experience more oxidative stress by monitoring the level of protein oxidation. Immunodetection of carbonylated proteins (Fig. 4C) demonstrated that proteins are more oxidized in *Δphx1* at late stationary phase. These results support a proposal that the actual mechanism by which Phx1 contributes to extend cell viability is through reducing ROS-mediated oxidative damage by inhibiting respiration in stationary phase.

### Extended lifespan of *Δpka1, Δsck2, or Δpyp1* mutants was compromised by *Δphx1* mutation

**Figure 5:**
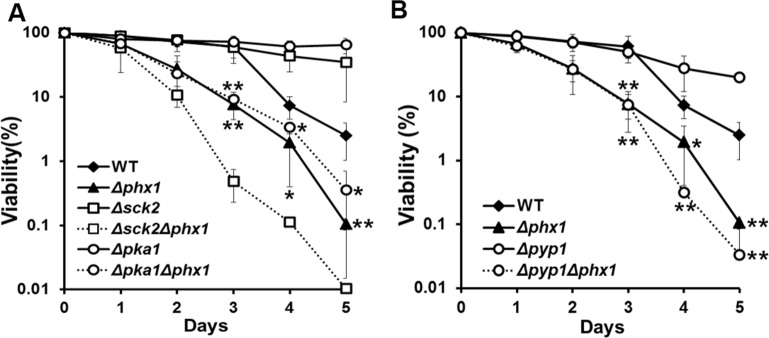
Extended lifespan of *Δpka1, Δsck2*, and *Δpyp1* mutants depends on Phx1 function. (**A**) Viability of wild type (972), *Δphx1* (JY01), *Δsck2* (JY06), *Δsck2Δphx1* (JY08), *Δpka1* (JY05), and *Δpka1Δphx1* (JY07) mutants during stationary phase. Each strain was grown to stationary phase in complex (YE) media, and examined viability on solid YES plates. At least three independent experiments were carried out to obtain survival curves for each strain. * and ** represent P-values of <0.1 and <0.05, respectively, for differences between wild type and *Δphx1* or *Δpka1Δphx1* at days 3 to 5. (**B**) Viability of wild type (972), *Δphx1* (JY01), *Δpyp1* (JY09; Sty1 constitutively activated), and *Δpyp1Δphx1* (JY10) was measured as in (**A**). * and ** represent P-values of <0.1 and <0.05, respectively, for differences between wild type and *Δphx1* or *Δpyp1Δphx1* following days 3 to 5.

In *S. pombe*, it has been demonstrated that nutrient signaling via serine/threonine kinases Pka1 and Sck2 has strong pro-aging effects, and deletion of either the *pka1* or *sck2* gene extends chronological lifespan [4]. We examined whether Phx1 has any genetic relation with Pka1 or Sck2, by examining long-term survival of *Δsck2Δphx1* and *Δpka1Δphx1* double deletion strains. The *Δsck2* and *Δpka1* mutants grown in complex media demonstrated extended long-term survival as previously reported [4, 6]. Introduction of the *Δphx1* mutation to these long-lived strains compromised their survival significantly (Fig. 5A). The stronger effect of the *phx1* mutation in the *Δsck2* mutant background suggests that in the absence of the Tor kinase, Phx1 may contribute more to cell survival than in the absence of protein kinase A. Constitutive activation of the Sty1 MAP kinase pathway of *S. pombe* has also been reported to extend chronological lifespan [6]. We examined whether the extended lifespan of a *Δpyp1* mutant, where Sty1 is constitutively activated by absence of inactivating phosphatase, is affected by the *phx1* mutation. Fig. 5B demonstrated that the prolonged survival phenotype of *Δpyp1* was greatly compromised by *Δphx1*, since the double mutant showed a similarly reduced survival as the *Δphx1* single mutant. Thus, Phx1 could be involved in mediating longevity in association with all three major pathways known to be involved in chronological lifespan.

## DISCUSSION

Pyruvate decarboxylase enzyme activities have been primarily studied in relation with ethanol fermentation. In our study, its specific function during stationary phase by diverting pyruvate from mitochondrial respiration to enhance chronological lifespan has been demonstrated. Unlike in *S. cerevisiae*, where the two paralogs Pdc1 and Pdc5 are engaged in ethanol fermentation throughout the growth phases, *S. pombe* produces phase-specific Pdcs; an exponential-specific Pdc101 and stationary-specific Pdc201 and Pdc202. High-throughput RNA sequencing and high-density tiling array analyses coincide with what we observed [28]. According to the array analyses, the *pdc101^+^* mRNA level was high during exponential phase and initial meiotic process, but decreased at quiescent phase, under stress conditions, and in late meiosis. The *pdc201^+^* mRNA level showed strong induction at quiescent phase, under stress conditions, and in meiosis, especially during the first and the third meiotic processes including conjugation and S phase. The *pdc202^+^* mRNA level dramatically increased during the late meiotic process in forming spores [20]. The *pdc102^+^* mRNA was rarely detected in most conditions examined. Recent quantitative proteome data also show a similar picture [29]: Pdc101 is expressed at ~500,000 copies per cell during growth but at only at ~200,000 copies during quiescence, whereas Pdc201 increases from ~7000 copies per cell during growth to ~52,000 copies during quiescence; Pdc102 and Pdc202 have not been detected at the protein level.

How this difference in Pdc expression is related with difference in sugar metabolism between budding and fission yeasts is an intriguing question. Comparative genomic work [30] provided some explanations for a glucose-dependent life style of fission yeasts. The gene content verifies the observation that fission yeast cannot utilize ethanol as a sole carbon source unlike budding yeasts [31]. Regulation of energy metabolism in response to glucose depletion is also somewhat different between budding and fission yeasts. Whereas respiratory genes for oxidative phosphorylation or the TCA cycle are induced on glucose depletion as observed for *S. cerevisiae*, expression of genes for the pyruvate dehydrogenase complex and *adh1^+^*is reduced, so that efficient use of pyruvate for respiration is thought to be prevented upon glucose depletion [30]. In our study, we demonstrated that Pdc201 and Pdc202 proteins are elevated upon glucose depletion to inhibit respiration.It has been reported that Crabtree-positive yeasts such as *S. cerevisiae* and *S. pombe* undergo aerobic ethanol fermentation, and have higher levels of Pdc enzyme activity, which is further induced by a glucose pulse [32]. In contrast to induction of Pdc1, Pdc5, and Pdc6 by a glucose pulse in *S. cerevisiae*, the stationary-phase specific PDC genes in *S. pombe* are repressed by a glucose pulse, and only the exponential-specific *pdc101^+^* gene is induced (data not shown). Therefore, the differential expression and characteristics of PDCs in *S. pombe* may underlie the difference in sugar and energy metabolism between fission and budding yeasts.

**Figure 6:**
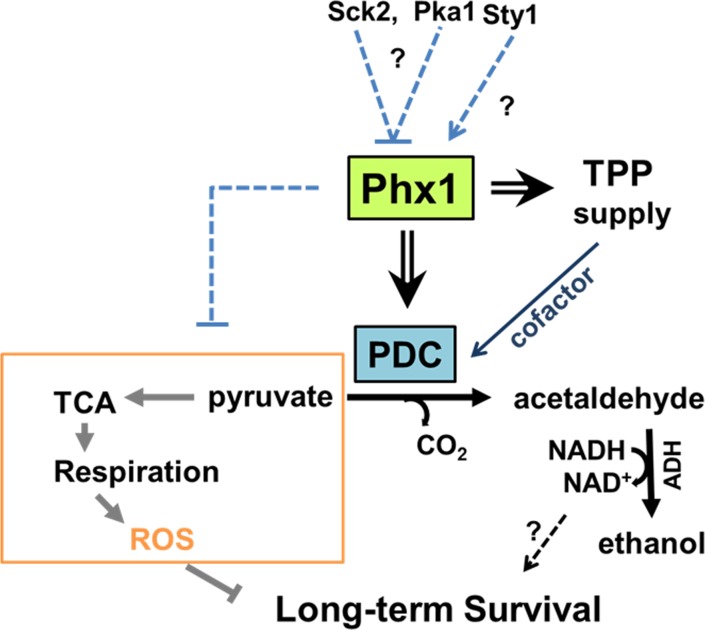
Scheme for mechanism of Phx1 to prolong viability during stationary phase Phx1 increases the long-term (stationary) viability of *S. pombe* cells via increasing stationary phase-specific pyruvate decarboxylase activities (PDC) through increased synthesis of Pdc201 and Pdc202 proteins and TPP supply (synthesis and transport). Elevation of stationary phase-specific PDC decreases the amount of pyruvate available for TCA cycle and respiration, which generates ROS that inhibits long-term survival during the stationary phase. Overall effects of Phx1 on the respiratory pathway (orange block) are presented with a dashed line. Whether increased production of NAD^+^ during fermentation contributes to long-term survival is not certain. There is a possibility that Phx1 is regulated negatively by the nutrient-dependent Pka1 and Sck2 kinases, and positively by the stress-activated MAP kinase Sty1. Arrows and cross bars indicate activation and repression, respectively.

In this study, we demonstrated Phx1-dependent induction of Pdc201 and Pdc202 during stationary phase and proposed that a mechanism by which Phx1 supports long-term survival is partly achieved by shifting metabolic energy flux away from respiration towards fermentation by regulating pyruvate node. As demonstrated in a simplified scheme in Fig. 6, this mechanism could bring forth at least two effects; one to reduce ROS formation generated from respiration (effect I) and the other to increase the level of ethanol and NAD^+^/NADH (effect II). Effect I has been validated in this study, by showing that the *Δphx1* mutant is elevated in respiration and ROS production during stationary phase. Contribution of effect II to long-term survival awaits further investigation. Previous studies with exponentially growing *S. cerevisiae* reported that metabolic reconfiguration occurred to cope with oxidative stress by inhibiting glycolysis and elevating the pentose phosphate pathway, which is anti-oxidative, via rapid modulation of proteins and gene expression [33]. Our work reveals another distinct way of redirecting metabolism through pyruvate decarboxylase (PDC) from respiration to fermentation upon glucose depletion, by which fungal cells could ensure long-term survival in stationary phase. This strategy could provide a way of coping with oxidative stress encountered during the stationary phase. Our observation that Phx1-mediated metabolic changes eventually contributed to long-term survival by curtailing production of ROS and the subsequent oxidative damage in the stationary phase coincides with previous reports on the damaging effect of ROS on chronological lifespan [34]. Previous observations that caloric restriction by low glucose enhances respiration, which in turn elevates lifespan without employing anti-oxidative measures, appear contradictory to what we observe in this study [11]. Actually they are not. What we report is a consequence of glucose limitation in post-mitotic cells after cells entered stationary phase, in contrast to growth on limited glucose during exponential growth. In our study, we presented evidence that stationary-phase specific elevation of PDC enzymes contributed to a decrease in respiration and in ROS generation and subsequent oxidative damage when cells entered the stationary phase. This is a distinct mechanism that also differs from caloric restriction-mediated increase in respiration and ROS generation during the exponential phase to activate the Sty1 MAPK pathway to enhance anti-stress gene expression for stationary phase survival [6, 9].

Conversion of pyruvate to acetaldehyde by PDC is followed by the action of alcohol dehydrogenase (ADH) to produce ethanol along with NAD^+^. There are reports that overexpression of the *adh1^+^* gene extends lifespan in *S. cerevisiae* [35] and in *S. pombe* [36]. How ADH increases lifespan is not certain. NAD^+^ can be speculated to activate Sir2, an NAD^+^-dependent histone deacetylase, which is known as an anti-aging factor in many eukaryotic systems from *S. cerevisiae* to mammals [37]. Therefore, whether the elevation of fermentation during stationary phase can serve as a way to increase lifespan in NAD^+^-dependent way in *S. pombe* is an interesting question that deserves further studies in the future.

How stationary phase-specific induction of Phx1 activity is regulated in *S. pombe* is an intriguing question. We observed that the extended lifespans of *Δsck2*, *Δpka1*, and *Δpyp1* mutants are all diminished by introducing the *Δphx1* mutation, to similar or even lower levels than for the *Δphx1* single mutant (Fig. 5). This finding raises the possibility that Phx1 acts downstream of the Pka1 and Tor/Sck2 pathways of nutrient signaling, being negatively regulated by these kinases. However, the limited information does not support any particular mode of signal transduction. Phx1 could be activated in synthesis and/or activity by components of TOR, PKA, and MAPK pathways. On the other hand, there is a possibility that Phx1 is not regulated by any of these signal transduction pathways, but performs a critical function whose absence abolishes the life-sustaining benefits of deleting proaging kinases or constitutive activating a stress kinase. The amino acid sequence of Phx1 contains some candidate motifs for phosphorylation, recognizable by Sck2 and Pka1. Sck2 is a homolog of S6 kinase (S6K), which is a serine/threonine-specific protein kinase that functions in diverse cellular processes such as translational regulation, cell-cycle regulation, cell growth control, and cellular ageing related with TORC1 network [10]. S6K phosphorylation motif is known as RXRXXS/T, which overlaps with 14-3-3 binding motif RSXpSXP [38]. There are two S6K recognition motifs (residue 576-581 and 645-652) on Phx1 protein, the latter one overlapping with 14-3-3 protein binding motif (residue 645-652). Phx1 also carries a putative PKA target sequence (R-R-X-S/T-X) in residues 578-582. This region overlaps with an S6K motif. Investigation of regulatory mechanism for Phx1, along with its interaction with other signaling pathways, will reveal more complete picture for how post-mitotic cells maintain their viability.

## EXPERIMENTAL PROCEDURES

### Strains and culture media

*S. pombe* strains and plasmids we used in this study are listed in supplementary Table S3. The *Δpdc102, Δpdc201, Δpdc202, Δpka1, and Δsck2* deletion strains were obtained from the Bioneer mutant library version 2.0 (Bioneer Corporation, Korea), and verified by PCR for correct deletions. Through mating of Bioneer auxotrophic strains with 972 (*h^-^*), we isolated prototrophic *Δpdc201*(JY02), *Δpdc202* (JY03), *Δpdc102* (JY04), *Δpka1*(JY05) and *Δsck2* (JY06) mutants, following spore selection and confirmation by PCR. The prototrophic *Δpka1Δphx1* (JY07) and *Δsck2Δphx1* (JY08) double deletion mutants were obtained by transforming *Δpka1::kmx4* or *Δsck2::kmx4* cassette to prototrophic *Δphx1* (JY01) strain, following marker selection and confirmation by PCR. The *Δpdc201Δpdc202* (JY11) double mutant was obtained by introducing *Δpdc202::natMX6* cassette to the prototrophic *Δpdc201* (JY02) strain. All recombinant plasmids were confirmed by nucleotide sequencing. Growth and maintenance of *S. pombe* strains were generally done in Edinburgh minimal medium (EMM, 2% glucose) or in yeast extract medium (YE, 3% glucose) with appropriate supplements [15]. For conjugation and sporulation, malt extract medium (ME, 3% malt extract) was used. For liquid culture, cells were inoculated at initial OD_600_ of 0.02 and were routinely grown in EMM to OD_600_ of ~1 for exponential phase (~18 h of culture time) or OD_600_ of ~10 for stationary phase (~50 h of culture time) at 30°C with shaking at 180 rpm.

### Microarray analysis

We used DNA microarrays displaying probes for >99% of all known and predicted genes of *S. pombe* spotted in duplicate onto glass slides [39]. For RNA preparation, WT(JH43) and *Δphx1* (ESX5) cells were grown in EMM for 80 hours after inoculation at initial OD_600_ of 0.02. RNA extraction, hybridization and initial data processing and normalization were performed as previously described [39]. Four independent biological experiments were performed, including two dye swaps. The data were visualized and analyzed using GeneSpring (Agilent), and were deposited in ArrayExpress (E-MTAB-2285). The significance of overlaps between different gene lists was calculated in GeneSpring using a standard Fisher's exact test, and *P* values were adjusted with a Bonferroni multiple testing correction. Cut-off values of 2-fold change of the average value from four biological repeats. Gene annotations were downloaded from PomBase (http://www.pombase.org/).

### Quantitative real-time PCR (qRT-PCR)

Each RNA sample (1 μg/μl) was reverse-transcribed into cDNA using RevertAid™ Reverse Transcriptase kit (Fermentas). Each PCR was performed with SYBR Green/ROX qPCR master mix (Fermentas) and *act1*^+^ mRNA-specific primers were used for an internal control. Triplicate PCRs for gene-specific primer pairs of each gene were carried out according to manufacturer's instruction in a qRT-PCR machine(Stratagene MX3000P QPCR system, Agilent Technologies) with analysis software MXpro (Agilent Technologies). Real-time PCR data were analyzed by the comparative C_T_ method [40] to calculate fold changes.

### Analysis of thiamine and thiamine phosphates by HPLC

Cells were grown in liquid EMM at 30°C and harvested at different time points, followed by washing with distilled water. Thiamine and thiamine phosphates were extracted and determined by HPLC as described by Schweingruber AM *et al*. [17]. The thiamine mixture was injected into an HPLC column (a PRP-1 main column, 10 μm, 250X4.1 mm, Hamilton no.79427) in Varian Prostar HPLC system equipped with a fluorescence detector (excitation at 365 nm and emission at 430 nm). Thiamine and its derivatives were eluted with steep gradients of solvent A (8.5 mM sodium phosphate buffer, pH 8.5) and solvent B (methanol, HLPC-grade, Merck). Thiamine phosphates were eluted in the order of triphosphate, diphosphate and monophosphate at 10% B, followed by thiamine after steep gradient of 50% B. As standards, thiamine monophosphate (TMP) and thiamine pyrophosphate (TPP) (Sigma) were monitored in parallel.

### Enzyme activity assay

Pyruvate decarboxylase activity in cell extracts (20 μg total protein) was determined as described previously [41]. The rate of disappearance of absorption at 340 nm by NADH was monitored on Simadzu UV-1650pc spectrophotometer. The enzyme activity was presented as U/mg protein, where 1 unit will convert 1.0 μmole of pyruvate to acetaldehyde per minute at pH 6.0 at 25°C.

### Long-term survival assay

To measure cell viability during stationary phase, cells were inoculated to liquid EMM or YE media at initial OD_600_ of 0.02 and grown for about 40 h or 30 h until OD_600_ reached the maximum cell density of about 9 or 12, respectively. From this time point (day 0), aliquots were taken each day and plated on complex YES medium, followed by incubation at 30°C for 2 days for colony counting. The average % viabilities with standard deviations were assessed from three independent experiments.

### Measurement of oxygen consumption

The respiration rate was measured polarographically at 30°C by using an oxygen electrode probe in YSI5300A Biological Oxygen Monitor System (Yellow Spring Instrument). After collection of 0.5 ml of the cells grown in EMM at each time point and at indicated OD_600_ point by centrifugation at 3000 g, cells were suspended in 3 ml of 50 mM potassium phosphate (pH 6.5) with 0.1 M glucose. Cell suspension was introduced into the sample chamber and the amount of oxygen consumed was recorded for 15 minutes.

### Measurement of intracellular ROS level

Intracellular ROS levels were determined by using redox-sensitive fluorescent probe 2′, 7′-dichlorofluorescin diacetate (DCFH-DA, Sigma).To each aliquot of 0.5 ml cultured cells, DCFH-DA (50 mM in DMSO) was added to 50 μM (final), and incubated for 1 h at 30°C in the dark. The amount of ROS-oxidized DCF was analyzed by flow cytometry, using FACS Canto (Becton Dickinson) at low flow and monitoring in FITC-A channel (detecting green fluorescence).A total of 10,000 live cells for each sample were analyzed for DCF - dependent fluorescence.

### Measurement of protein carbonylation

Protein carbonylation was detected by OxyBlot^™^ protein oxidation detection kit as recommended by the manufacturer (Millipore). Cells were grown in EMM, and harvested at each time point during stationary phase. Protein extracts were prepared after vortexing harvested cells with glass beads in lysis buffer containing either 1-2% β-mercaptoethanol or 50 mM DTT. Following protein quantification by Bradford assay, each 5 μl aliquot containing 20 μg of protein was processed for DNP derivatization, electrophoresed on 10% SDS-PAGE, and immunodetected by anti-DNP antibody. As a loading control, a parallel electro-transferred membrane was stained with Ponceau S solution. The anti-DNP immunoblots and Ponceau S stained blots were scanned and quantified by chemiluminescence imaging system (Fusion solo, Vilber Lourmat) with Multi Gauge (Fuji) program.

## SUPPLEMENTARY TABLES AND FIGURES




