# Supplementary material for: A metabolic strategy to enhance long-term survival by Phx1 through stationary phase-specific pyruvate decarboxylases in fission yeast
Source: Aging (Albany NY). 2014 Jul 29;6(7):587–601. doi: 10.18632/aging.100682 (PMC4153625; doi:10.18632/aging.100682)
Supplement: Supplementary file 1 [file aging-06-587-s001.docx]

| **Table S1. List of repressed genes in *Δphx1* mutant^a^** | | | | | | | | |
| --- | --- | --- | --- | --- | --- | --- | --- | --- |
| **systematic name** | **gene name** | **Description** | | | **Mutant/WT Expression Ratio** | | **meiotic expression**  **phase** | **overlapped function** |
|  |  |  |  |  | **mean** | **±SD** |  |  |
| **thiamin and derivative biosynthetic process** | | | | | | | | |
| SPCC1223.02 | nmt1,  thi3 | no message in thiamine, nmt1, thi3 | | | 0.07 | 0.11 |  |  |
| SPBC26H8.01 | nmt2, thi2 | thiazole biosynthetic enzyme, thi2, nmt2 | | | 0.46 | 0.33 |  |  |
| **carbohydrate metabolic process** | | | | | | | | |
| **glycolysis** | | | | | | | | |
| SPBPB21E7.01c | eno1 | enolase, eno102, SPAP8B6.07c, SPBPB8B6.07c, SPAPB21E7.01c, eno1 | | | 0.11 | 0.07 |  |  |
| SPBC354.12 | gpd3 | glyceraldehyde 3-phosphate dehydrogenase Gpd3, gpd3 | | | 0.45 | 0.09 |  |  |
|  |  |  | | |  |  |  |  |
| **alcohol catabolic process/glycerol catabolic process** | | | | | | | | |
| SPAC977.16c | dak2 | dihydroxyacetone kinase Dak2 (PMID 9804990) | | | 0.19 | 0.03 | delayed |  |
|  |  |  | | |  |  |  |  |
| **alcohol catabolic process/pyruvate metabolic process** | | | | | | | | |
| SPAC13A11.06 | pdc202 | pyruvate decarboxylase | | | 0.08 | 0.06 | late |  |
| SPAC3G9.11c* | pdc201 | pyruvate decarboxylase (predicted) | | | 0.53 | 0.2 | continuous |  |
| **response to stress** | | | | | | | | |
| SPAC869.09 |  | conserved fungal protein | | | 0.14 | 0.07 | late |  |
| SPAC22H10.13 | zym1 | metallothionein Zym1 | | | 0.29 | 0.07 | early | di-, tri-valent inorganic cation homeostasis |
| SPAC22G7.11c |  | conserved fungal protein | | | 0.39 | 0.07 | late |  |
| SPBC8E4.05c |  | fumarate lyase superfamily | | | 0.45 | 0.02 |  |  |
| SPAC11D3.01c* |  | conserved fungal protein | | | 0.53 | 0.15 | late |  |
| SPAC7D4.04 | taf1 | Taz1 interacting factor 1 | | | 0.33 | 0.06 |  | autophagy/conjugation/telomere maintenance |
| SPBC3E7.02c | hsp16 | heat shock protein, hsp16 | | | 0.40 | 0.12 | middle | response to heat / under conditions of deoxyribonucleotide depletion and DNA damage |
| **transport** | | | | | | | | |
| SPBC839.06 | cta3 | P-type ATPase, calcium transporting Cta3 | | | 0.36 | 0.23 |  | di-, tri-valent inorganic cation homeostasis |
| SPAC977.17 |  | MIP water channel | | | 0.46 | 0.12 |  |  |
| SPAC17A2.01* | bsu1 | high-affinity import carrier for pyridoxine, pyridoxal, and pyridoxamine Bsu1 | | | 0.55 | 0.39 |  |  |
| SPAC17C9.16c | mfs1 | MFS family transmembrane transporter Mfs1 | | | 0.28 | 0.21 | transient |  |
| SPCPB1C11.03* |  | cysteine transporter (predicted) | | | 0.60 | 0.42 |  |  |
| **RNA metabolic process** | | | | | | | | |
| SPBPB21E7.07 | aes1 | enhancer of RNA-mediated gene silencing, aes1, SPAPB21E7.07 | | | 0.36 | 0.26 |  | chromatin silencing by small RNA |
| SPBC530.08 |  | membrane-tethered transcription factor (predicted) | | | 0.43 | 0 |  |  |
| **non-coding RNA** | | | | | | | | |
| SPNCRNA.26 | prl26 | non-coding RNA (predicted),poly(A)-bearing RNA | | | 0.26 | 0 |  |  |
| SPNCRNA.74 |  | antisense RNA (predicted) | | | 0.30 | 0.06 |  |  |
| SPNCRNA.101 |  | non-coding RNA (predicted) | | | 0.31 | 0.06 |  |  |
| SPNCRNA.73 |  | antisense RNA (predicted) | | | 0.34 | 0.04 |  |  |
| SPNCRNA.01 | prl01 | non-coding RNA (predicte),possibly part of the UTR of eta2 | | | 0.37 | 0.05 |  |  |
| SPNCRNA.79 |  | non-coding RNA (predicted) | | | 0.43 | 0.13 |  |  |
| SPNCRNA.12 | prl12 | non-coding RNA (predicted),poly(A)-bearing RNA | | | 0.47 | 0 |  |  |
| **vesicle-mediated transport** | | | | | | | | |
| **vesicle organization** | | | | | | | | |
| SPAC824.02 |  | GPI inositol deacylase | | | 0.32 | 0.08 |  | quality control and ER-associated degradation of GPI-anchored proteins |
| SPAC22E12.17c | glo3 | ARF GTPase activating protein | | | 0.37 | 0.03 |  | regulation of signaling pathway |
|  |  |  | | |  |  |  |  |
| **endocytosis** | | | | | | | | |
| SPAC3C7.02c |  | protein kinase inhibitor (predicted) | | | 0.39 | 0.23 | late | response to heat stress |
| SPCC4F11.04c | imt2 | mannosyltransferase complex subunit, | | | 0.49 | 0.14 | middle |  |
| **protein phosphorylation** | | | | | | | | |
| SPBC725.06c | ppk31, mug25 | serine/threonine protein kinase, ppk31 (*S.c.* rim15 homologue) | | 0.09 | | 0.01 |  | Has a role in meiosis. |
| SPBC19F8.07 | crk1 | cyclin-dependent kinase activating kinase Crk1, crk1, mop1, mcs6 | | 0.38 | | 0.02 | middle | cytokinesis |
| **cytoskeleton organization** | | | | | | | | |
| SPBC1289.14 |  | adducin N-terminal domain protein, , SPBC8E4.10c | | | 0.40 | 0.18 |  | response to stress |
| **oxidation-reduction** | | | | | | | | |
| SPBC1198.01 |  | glutathione-dependent formaldehyde dehydrogenase | | | 0.25 | 0.07 | late |  |
| SPAC27D7.12c | but1 | neddylation pathway protein But1 | | | 0.34 | 0 | middle | Has a role in meiosis, cell elongation |
| SPAC5H10.04* |  | NADPH dehydrogenase (predicted) | | | 0.5083 | 0.14 |  |  |
| **others** | | | | | | | | |
| SPAC869.06c |  | | HHE domain cation binding protein (predicted) | | 0.04 | 0.05 | late | mitochondria |
| SPAPB18E9.04c |  | | glycoprotein | | 0.07 | 0.07 |  | cell surface |
| SPBPB21E7.02c |  | | phosphoglycerate mutase family | | 0.07 | 0.03 |  |  |
| SPBPB21E7.04c |  | | human COMT ortholog 2,catechol O-methyltransferase | | 0.11 | 0.04 |  |  |
| SPAC1F7.06 |  | | ThiJ domain protein | | 0.16 | 0.03 | late |  |
| SPAPB18E9.03c |  | | dubious | | 0.17 | 0 |  |  |
| SPBC19C7.04c |  | | conserved yeast protein | | 0.24 | 0.26 | early |  |
| SPAC4F10.17 |  | | conserved fungal protein | | 0.29 | 0.06 | late |  |
| SPAC1093.01 |  | | PPR repeat protein(Pentatricopeptide repeat ) | | 0.33 | 0.1 |  |  |
| SPAC11D3.02c |  | | ELLA family acetyltransferase (predicted) | | 0.37 | 0 |  |  |
| SPAC15F9.01c |  | | sequence orphan,central kinetochore associated family protein | | 0.42 | 0.01 | late |  |
| SPAC9E9.01 |  | | dubious | | 0.45 | 0.09 |  |  |
| SPAC30D11.02c |  | | sequence orphan | | 0.46 | 0 |  |  |
| SPBC530.07c |  | | TENA/THI domain | | 0.47 | 0.07 |  |  |
| SPCPB16A4.06c |  | | dubious | | 0.48 | 0.02 |  |  |
| SPACUNK4.12c | mug138 | | metallopeptidase | | 0.48 | 0.02 | middle | congugation |
| SPCC417.12 |  | | carboxylesterase-lipase family | | 0.49 | 0 | middle |  |
| SPAC1142.01* |  | | conserved eukaryotic protein, , SPAC17G6.18 | | 0.51 | 0.14 |  |  |

^a^ Transcripts whose level decreased more than 2-fold in the mutant than in the wild type were presented. Average values from four biological replicates were used to apply cutoff. The genes whose expression changed more than 2-fold in three out of four replicate experiments, with average values slightly exceeding 0.5, were included and marked (*).

| **Table S2. List of induced genes in *Δphx1* mutant^a^** | | | | | | |
| --- | --- | --- | --- | --- | --- | --- |
| **systematic name** | **gene name** | **Description** | **Mutant/WT Expression Ratio** | | **meiotic expression**  **phase** | **overlapped function** |
|  |  |  | **mean** | **±SD** |  |  |
| **carbohydrate & energy metabolism** | | | | | | |
| **oligosaccharide catabolic process** | | | | | | |
| SPAPB24D3.10c | agl1 | alpha-glucosidase Agl1, | 18.84 | 5.0 | late |  |
| SPCC191.11 | inv1 | beta-fructofuranosidase, | 11.32 | 5.2 |  |  |
| SPAC1039.11c |  | alpha-glucosidase, SPAC922.02c | 2.30 | 0.3 | middle |  |
|  |  |  |  |  |  |  |
| **tricarboxylic acid metabolic process & mitochondria related** | | | | | | |
| SPCC191.07 | cyc1 | cytochrome c, cyc1 | 2.60 | 0.6 |  |  |
| SPAC6C3.04 | cit1 | citrate synthase | 2.37 | 0.5 |  |  |
| SPAC24C9.06c | aco1 | aconitate hydratase | 2.32 | 0.2 |  |  |
|  |  |  |  |  |  |  |
| **NADH oxidation** | | | | | | |
| SPAC3A11.07 |  | NADH dehydrogenase | 2.21 | 1.3 |  |  |
|  |  |  |  |  |  |  |
| **pentose-phosphate shunt, oxidative branch/NADPH regeneration** | | | | | | |
| SPAC26H5.09c |  | gfo/idh/mocA family oxidoreductase (predicted) | 3.34 | 0.0 |  |  |
| SPAC3C7.13c |  | glucose-6-phosphate 1-dehydrogenase, | 2.16 | 0.8 | continuous | cellular response to stimulus |
| SPAC3A12.18 | zwf1 | glucose-6-phosphate 1-dehydrogenase, SPAC9.01 | 2.05 | 0.9 |  | cellular response to stimulus |
| SPAC4G9.12 |  | gluconokinase | 3.02 | 0.6 | middle |  |
|  |  |  |  |  |  |  |
| **monocarboxylic acid metabolic process** | | | | | | |
| SPACUNK4.10 |  | 2-hydroxyacid dehydrogenase,glyoxylate reductase (predicted) | 3.90 | 2.9 |  |  |
|  |  |  |  |  |  |  |
| **alcohol metabolic process** | | | | | | |
| SPCC1223.03c | gut2 | glycerol-3-phosphate dehydrogenase Gut2, | 2.55 | 1.1 | delayed | cellular response to stimulus |
| SPAC9E9.09c |  | aldehyde dehydrogenase (predicted) | 2.44 | 1.1 |  | cellular response to stimulus |
| SPAC25B8.03 | psd2 | phosphatidylserine decarboxylase | 2.14 | 1.1 |  |  |
| SPBC1773.05c | tms1 | hexitol dehydrogenase | 2.08 | 0.5 | late | cellular response to stimulus |
| SPAC630.08c | erg25 | C-4 methylsterol oxidase | 2.07 | 1.5 |  |  |
|  |  |  |  |  |  |  |
| **nucleoside metabolic process** | | | | | | |
| SPCC191.05c |  | nucleoside 2-deoxyribosyltransferase | 2.54 | 1.2 | transient |  |
| SPBC800.11 |  | inosine-uridine preferring nucleoside hydrolase | 2.11 | 0.5 | transient |  |
| **response to stress** | | | | | | |
| SPAC343.12 | rds1 | conserved fungal protein, rds1 | 7.39 | 5.3 | late |  |
| SPAP14E8.02 | tos4 | FHA domain protein Tos4 (predicted) | 4.15 | 0.0 |  | response to DNA damage stress |
| SPBC660.05 |  | hypothetical protein | 3.12 | 1.7 | continuous |  |
| SPBC1271.08c |  | sequence orphan | 3.07 | 1.3 |  |  |
| SPCC1739.08c |  | short chain dehydrogenase | 3.02 | 2.2 | continuous |  |
| SPAC20G4.03c | hri1 | eIF2 alpha kinase Hri1, hri1 | 2.19 | 1.2 |  | response to osmotic stress |
| SPACUNK4.15 |  | 2',3'-cyclic-nucleotide 3'-phosphodiesterase, | 2.08 | 1.1 | middle |  |
| SPAC27D7.11c |  | But2 family protein | 2.02 | 0.6 |  |  |
|  |  |  |  |  |  |  |
| **cellular response to oxidative stress** | | | | | | |
| SPAC821.10c | sod1 | superoxide dismutase Sod1 | 2.25 | 1.2 |  |  |
| SPBC106.02c | srx1 | sulphiredoxin | 2.20 | 0.6 |  |  |
| SPAC11D3.16c |  | sequence orphan | 2.09 | 1.5 | transient |  |
| SPAPB1A10.12c | alo1 | D-arabinono-1,4-lactone oxidase | 2.04 | 0.3 |  |  |
| **conjugation / meiosis** | | | | | | |
| SPAC23E2.03c | ste7 | meiotic suppressor protein Ste7 | 4.82 | 4.7 | continuous |  |
| SPAPB8E5.05 | mfm1 | M-factor precursor | 4.57 | 2.9 | delayed |  |
| SPAC22F3.12c | rgs1 | regulator of G-protein signaling Rgs1 | 4.18 | 2.5 | delayed | cellular response to stimulus |
| SPBC29B5.02c | isp4 | OPT oligopeptide transporter family | 4.12 | 2.3 | continuous | cellular response to stimulus |
| SPAC27D7.03c | mei2 | RNA-binding protein involved in meiosis Mei2 | 3.76 | 2.0 | delayed |  |
| SPAC513.03 | mfm2 | M-factor precursor | 3.55 | 2.0 | delayed |  |
| SPBPJ4664.03 | mfm3 | M-factor precursor | 3.14 | 1.0 | delayed |  |
| SPCC1795.06 | map2 | P-factor | 3.10 | 0.0 | delayed |  |
| SPAC25B8.13c | isp7 | 2 OG-Fe(II) oxygenase superfamily | 2.47 | 0.1 | transient | cellular response to stimulus |
| SPBC32C12.02 | ste11, aff1, stex | transcription factor Ste11 | 2.23 | 1.1 | delayed |  |
| SPAC22A12.07c | ogm1, oma1 | protein O-mannosyltransferase Ogm1 | 2.05 | 1.2 |  | cellular response to stimulus |
| SPBC2D10.06 | rep1, rec16 | MBF transcription factor complex subunit Rep1 | 2.03 | 0.0 | early |  |
| SPBC25B2.02c | mam1 | M-factor transporter Mam1 | 2.85 | 2.2 | delayed | mating |
| **transport** | | | | | | |
| **hexose transport** | | | | | | |
| SPAC1F8.01 | ght3 | hexose transporter | 4.04 | 2.6 | early | cellular response to stimulus |
| SPBC1683.08 | ght4 | hexose transporter | 2.98 | 2.1 | early | cellular response to stimulus |
| SPCC1235.14 | ght5 | hexose transporter | 2.35 | 1.5 | middle |  |
| SPCC548.06c | ght8 | hexose transporter | 2.13 | 1.4 | middle |  |
|  |  |  |  |  |  |  |
| **iron assimilation** | | | | | | |
| SPAC1F7.07c | fip1 | iron permease | 4.35 | 1.5 |  |  |
| SPAC1F7.08 | fio1 | iron transport multicopper oxidase | 2.75 | 1.4 |  |  |
| SPBC947.05c |  | ferric-chelate reductase | 2.58 | 1.6 |  |  |
|  |  |  |  |  |  |  |
| **peptide transport** | | | | | | |
| SPBC13A2.04c | ptr2 | PTR family peptide transporter | 2.17 | 0.2 |  |  |
|  |  |  |  |  |  |  |
| **other transport** | | | | | | |
| SPBC16A3.02c |  | mitochondrial peptidase | 2.11 | 0.8 | continuous | cellular response to stimulus |
| SPCC794.03 |  | amino acid permease family | 4.13 | 2.5 |  |  |
| SPCC569.05c |  | spermidine family transporter | 3.73 | 1.6 |  | cellular response to stimulus |
| SPBC1348.05 |  | MFS family membrane transporter | 3.47 | 1.5 |  |  |
| SPAC750.02c |  | MFS family membrane transporter | 3.08 | 1.2 | late |  |
| SPBPB2B2.16c |  | MFS family membrane transporter | 2.88 | 0.7 |  |  |
| SPAC323.07c |  | MatE family transporter | 2.70 | 3.4 | transient |  |
| SPAC1B3.16c | vht1 | vitamin H transporter Vth1 | 2.48 | 0.5 |  |  |
| SPCC18B5.01c | bfr1, hba2 | brefeldin A efflux transporter, SPCPJ732.04c | 2.36 | 1.0 |  | cellular response to stimulus |
| SPBC530.10c | anc1 | mitochondrial adenine nucleotide carrier Anc1 | 2.36 | 1.4 |  |  |
| SPAC1610.03c | crp79, meu5 | poly(A) binding protein Crp79, mRNA export from nucleus | 2.31 | 0.0 | middle |  |
| SPBC530.02 |  | MFS family membrane transporter, | 2.11 | 1.1 |  |  |
| SPBC359.05 | abc3 | iron-regulated vaculoar ABC type transporter | 2.09 | 1.1 |  |  |
| SPAC222.12c | atp2 | F1-ATPase beta subunit, | 2.04 | 0.8 |  |  |
| **non-coding RNA** | | | | | | |
| SPNCRNA.03 | prl3 | non-coding RNA, poly(A)-bearing RNA (predicted) | 4.83 | 2.0 |  |  |
| SPNCRNA.93 |  | non-coding RNA (predicted) | 2.82 | 0.0 |  |  |
| SPNCRNA.133 |  | non-coding RNA (predicted) | 2.75 | 1.0 |  |  |
| SPNCRNA.134 |  | non-coding RNA (predicted) | 2.55 | 0.8 |  |  |
| SPNCRNA.07 | meu3, prl7 | non-coding RNA Meu3 | 2.25 | 0.1 |  |  |
| SPNCRNA.63 |  |  | 2.04 | 0.3 |  |  |
| **others** | | | | | | |
| SPAC186.05c |  | hypothetical protein, | 18.17 | 2.8 |  |  |
| SPAC977.07c |  | cell surface glycoprotein (predicted) | 7.59 | 0.0 |  |  |
| SPAC186.04c |  | pseudogene, similar to N-terminal of transmembrane channel | 5.74 | 1.7 | continuous |  |
| SPAC1A6.03c |  | phospholipase, | 4.66 | 2.9 |  | glycerophospholipid catabolic process |
| SPBC660.14 | mik1 | mitotic inhibitor kinase Mik1, | 4.41 | 0.0 | middle | phosphate metabolic process |
| SPAC212.03 |  | hypothetical protein, | 4.18 | 0.0 |  |  |
| SPAPB18E9.05c |  | dubious | 3.74 | 0.7 |  |  |
| SPAC977.04 |  | pseudogene | 3.63 | 1.5 |  |  |
| SPAC977.05c |  | conserved fungal protein, | 3.31 | 1.4 |  |  |
| SPAC977.02 |  | S. pombe specific 5Tm protein family | 3.07 | 0.6 |  |  |
| SPCC584.16c |  | sequence orphan, | 2.99 | 1.0 |  |  |
| SPAC513.04 |  | sequence orphan | 2.74 | 0.6 |  |  |
| SPBC359.06 |  | adducin N-terminal domain protein, | 2.70 | 1.5 | early |  |
| SPAPB15E9.01c |  | glycoprotein, , SPAPB18E9.06c | 2.67 | 0.2 |  |  |
| SPBC1348.04 |  | methyltransferase | 2.65 | 0.5 |  |  |
| SPAC977.03 |  | methyltransferase | 2.60 | 0.6 |  |  |
| SPBC9B6.03 |  | zinc finger protein, | 2.53 | 1.3 | transient |  |
| SPCC553.10 |  | glycoprotein, | 2.46 | 0.7 |  | cell adhesion |
| SPCC1450.07c |  | D-amino acid oxidase,FAD dependent oxidoreductase | 2.30 | 0.8 | transient |  |
| SPBPB2B2.19c |  | S. pombe specific 5Tm protein family | 2.29 | 0.1 | late |  |
| SPBC1348.03 |  | S. pombe specific 5Tm protein family | 2.28 | 1.2 |  |  |
| SPBC1773.14 | arg7 | argininosuccinate lyase, | 2.26, | 0.1 |  | glutamine family amino acid biosynthetic process |
| SPAC977.01 |  | S. pombe specific 5Tm protein family | 2.22 | 0.2 | late |  |
| SPAC27E2.04c |  | dubious | 2.14 | 0.0 |  |  |
| SPAC24C9.08 |  | vacuolar carboxypeptidase (predicted) | 2.11 | 0.8 |  | vacuolar protein catabolic process |

^a^ Transcripts whose level increased more than 2-fold in the mutant than in the wild type were presented. Average values from four biological replicates were used to apply cutoff. The genes whose expression changed more than 2-fold in three out of four replicate experiments, with average values slightly exceeding 0.5, were included and marked (*).

| **Table S3. Strains and plasmids used in this study** | | |
| --- | --- | --- |
| **strains** | **genotype** | **source / ref.** |
| 972 | *h^-^* | Lab. Collection |
| ED665 | *h^-^ ade6-M210 leu1–32 ura4-D18* |  |
| JH43 | *h^-^ ade6-M210 leu1–32* |  |
| ESX5 | *h^-^ ade6-M210 leu1–32 ura4-D18 Δphx1::ura4+* | Kim *et al*. [1] |
| JY01 | *h^-^ ura4-D18 Δphx1::ura4+* |  |
| JY02 | *h^-^ Δpdc201::kanMX4* | This work |
| JY03 | *h^-^ Δpdc202::kanMX4* |  |
| JY04 | *h^-^ Δpdc102::kanMX4* |  |
| JY05 | *h^-^ Δpka1::kanMX4* |  |
| JY06 | *h^-^ Δsck2::kanMX4* |  |
| JY07 | *h^-^ ura4-D18Δpka1::kanMX4 Δphx1::ura4+* |  |
| JY08 | *h^-^ ura4-D18Δsck2::kanMX4 Δphx1::ura4+* |  |
| JY09 | *h^-^ Δpyp1::kanMX4* |  |
| JY10 | *h^-^ ura4-D18Δpyp1::kanMX4Δphx1::ura4+* |  |
| JY11 | *h^-^ Δpdc201::kanMX4 Δpdc202::natMX6* |  |
| **Plasmids** | **Description** | **Source/ref.** |
| pAEP1 | pREP1 based, adh1 promoter, leu1^+^ | Kwon *et al*. [2] |
| pAEP1-*pdc201^+^* | pAEP1 + *pdc201^+^* ORF (NdeI/BamHI) | This work |
| pAEP1-*pdcB202^+^* | pAEP1 + *pdcB202^+^* ORF (NdeI/BamHI) | This work |

[1] Kim JY, Kwon ES, Roe JH (2012) A homeobox protein Phx1 regulates long-term survival and meiotic sporulation in Schizosaccharomyces pombe. BMC microbiology 12: 86, [2] Kwon ES, Jeong JH, Roe JH (2006) Inactivation of homocitrate synthase causes lysine auxotrophy in copper/zinc-containing superoxide dismutase-deficient yeast Schizosaccharomyces pombe. The Journal of biological chemistry 281: 1345-1351
