# Supplementary material for: A metabolic strategy to enhance long-term survival by Phx1 through stationary phase-specific pyruvate decarboxylases in fission yeast
Source: Aging (Albany NY). 2014 Jul 29;6(7):587–601. doi: 10.18632/aging.100682 (PMC4153625; doi:10.18632/aging.100682)
Supplement: Supplementary file 2 [file aging-06-587-s002.pdf]

SUPPLEMENTARY DATA

Please browse the full text version of this manuscript to see Supplementary Tables 1-3.

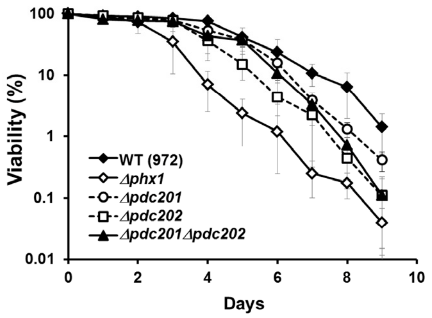

Figure S1. Effect of double mutations of pdc201 and pdc202 on long-term survival. Survival of the Δpdc201Δpdc202 double mutant (JY11) was monitored along with those of the wild type (972), *Δphx1* (JY01*), Δpdc201* (JY02), and *Δpdc202* (JY03), as described in Fig. 3. Average values with standard deviations from six independent experiments were presented for the double mutant. Values from three independent experiments were presented for the wild type and single mutants.
